# A home monitoring program including real-time wireless home spirometry in idiopathic pulmonary fibrosis: a pilot study on experiences and barriers

**DOI:** 10.1186/s12931-018-0810-3

**Published:** 2018-05-29

**Authors:** C. C. Moor, M. Wapenaar, J. R. Miedema, J. J. M. Geelhoed, P. P. Chandoesing, M. S. Wijsenbeek

**Affiliations:** Department of Respiratory Medicine, Erasmus University Medical Center, ‘s-Gravendijkwal 230, Rotterdam, 3015 CE The Netherlands

**Keywords:** Idiopathic pulmonary fibrosis, eHealth, Home monitoring, Spirometry

## Abstract

In idiopathic pulmonary fibrosis (IPF), home monitoring experiences are limited, not yet real-time available nor implemented in daily care. We evaluated feasibility and potential barriers of a new home monitoring program with real-time wireless home spirometry in IPF. Ten patients with IPF were asked to test this home monitoring program, including daily home spirometry, for four weeks. Measurements of home and hospital spirometry showed good agreement. All patients considered real-time wireless spirometry useful and highly feasible. Both patients and researchers suggested relatively easy solutions for the identified potential barriers regarding real-time home monitoring in IPF.

## Introduction

Idiopathic pulmonary fibrosis (IPF) is a progressive, devastating disease with a poor prognosis [1]. Symptoms as increasing shortness of breath and immobility make regular hospital visits a challenge for many patients. New eHealth technologies hold great potential for research and care by facilitating real-time, frequent data collection at home. In IPF, home monitoring experiences are limited and not yet implemented in daily care. Few studies using daily handheld spirometry have been performed in patients with IPF [2, 3]. These studies showed that home spirometry in IPF is feasible, may allow for better disease prediction and decrease sample size for future trials [2, 3]. However, earlier studies using home spirometry in interstitial lung diseases used paper-based collection or central read-out of Forced Vital Capacity (FVC) results [2–4]. This limits possibilities to control quality of measurements, or respond directly to FVC decline or non-adherence.

We assessed feasibility of a pre-developed home monitoring program in IPF [5], integrated with real-time, wireless home spirometry. Furthermore, we evaluated potential barriers and solutions for implementation of wireless home spirometry in this mostly elderly patient population.

## Methods

**Fig. 1:**
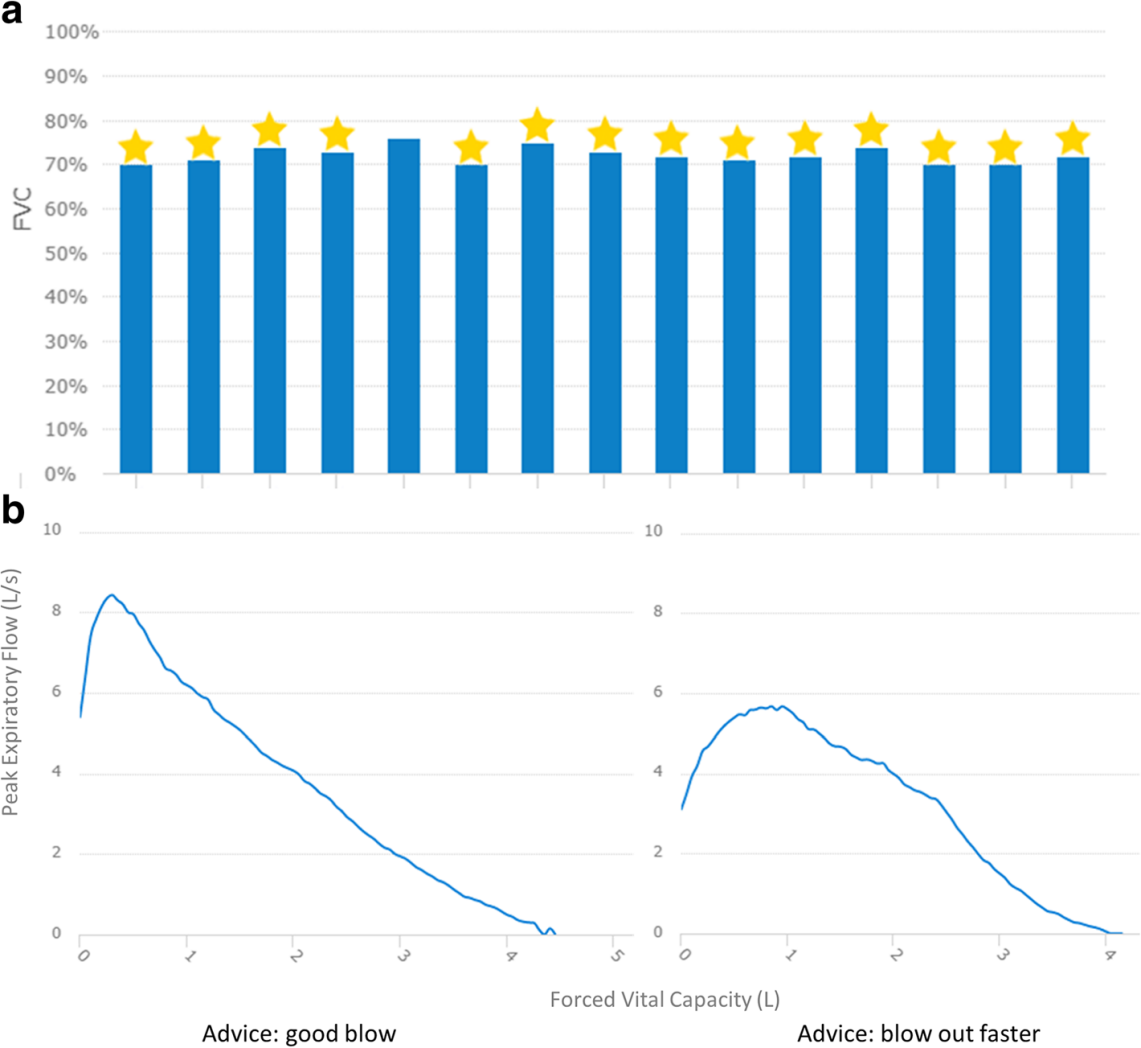
**a** Daily FVC in % predicted of one patient during two weeks. A star on top of the bar corresponds with a forced expiration > 6 s, and is intended as extra motivation for patients. **b** Two examples of flow volume loops including daily remarks/advices

This was a prospective pilot study at the Erasmus Medical Center in 2017. Consecutive outpatients with IPF were invited to participate. Approval of the Medical ethics committee was obtained, and participants provided written informed consent. Patients were asked to test the home monitoring program “IPF-online” (http://www.ipfonline.nl) for four weeks on a tablet. IPF-online is a secured online personal platform, following European safety regulations. The program consists of daily home spirometry, online patient-reported outcomes (PROs) at baseline and after four weeks, weekly reporting of side-effects and symptoms on visual analogue scales, an information library, medication coach and eConsultations. The bluetooth-enabled spirometer (MIR Spirobank Smart, Italy) transmits data real-time via a secure encrypted connection, enabling patients and healthcare providers to access data directly (Fig. 1). The system generates email alerts when patients report bothersome side-effects or FVC declines > 10% for three consecutive days. If patients fail to perform spirometry or record symptoms, they receive a reminder. Incorporated PROMs are King’s Brief Interstitial Lung Disease health status questionnaire, Hospital Anxiety and Depression Scale, Euroqol 5D-5 L and an evaluation questionnaire [6–8]. At start, patients received standardized instructions about the correct use of home spirometry and the different components of the online tool. Patients were considered trained when they were able to perform three good, reproducible FVC measurements, with less than 150 ml difference in the two highest FVCs. Before start of the study, potential barriers of the system were identified based on literature and own experiences. At baseline, potential barriers were discussed with patients. After four weeks, their experiences and suggestions were evaluated. Furthermore, patients performed hospital spirometry at baseline and after four weeks.

Pearson correlation and Bland-Altman plots were used to compare home with hospital spirometry, Wilcoxon signed ranked test was used to compare baseline with follow-up scores. Data are presented as mean (SD) or median (range).

## Results

Of 12 patients invited to participate, 10 patients were included (9 men), with a mean age of 71 years (5). All patients were on disease-modifying medication (60% nintedanib, 40% pirfenidone). The mean FVC was 3.28 L (1.04) or 79% of predicted (16).

### Reliability of home spirometry

**Fig. 2:**
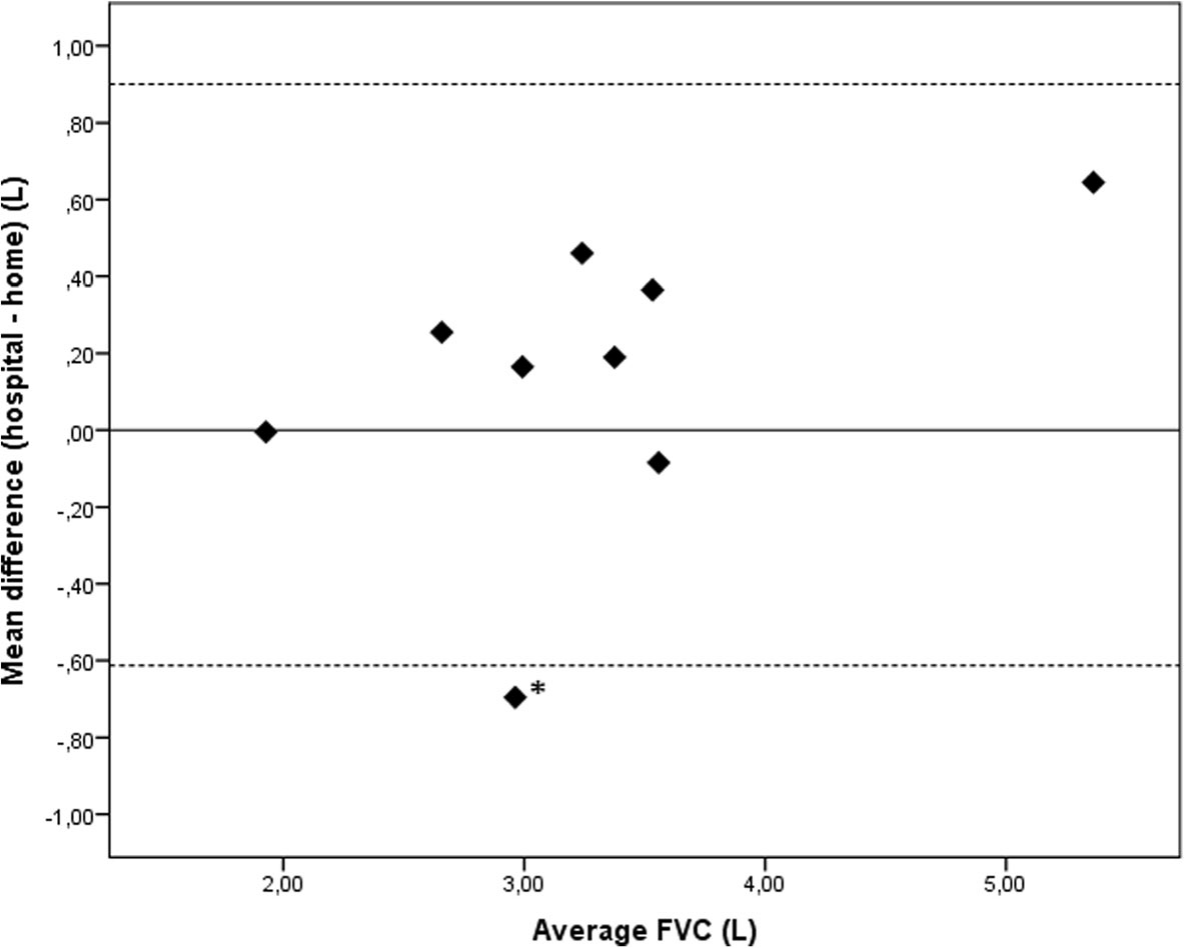
Bland-Altman plot comparing hospital and home spirometry. The value for hospital FVC is the mean of the hospital-based FVC at baseline and after four weeks. The value for home spirometry is the mean of 28 home FVC readings. The solid line represents the mean difference and the dashed lines 95% limits of agreement (− 0.61 to 0.90 L).* This patient did not use the mouthpiece correctly leading to more variable and higher readings compared to hospital spirometry

Measurements of home and hospital spirometry for FVC (*r* = 0.94 (*p* < 0.001)) and FEV1 (*r* = 0.97 (*p* < 0.001)) were highly correlated, and a Bland-Altman plot showed good agreement (Fig. 2). Median difference between hospital and home spirometry was 0.22 L (0.01-0.69 L) with overall lower readings for home spirometry. To evaluate within-subject reproducibility, the median SD for 28 measurements was calculated (0.13 L (0.05 -0.39 L)). The median coefficient of variation was 3.76% (3-12%).

### Feasibility and potential barriers of home spirometry in patients with IPF

The vast majority of patients considered daily spirometry easy (80%) and not burdensome at all (90%), the other patients were neutral. The mean adherence to home spirometry was 98.8% (2.5). Most patients (80%) found it pleasant to see their FVC results, 20% was neutral. All patients considered real-time spirometry useful and would recommend it to others, 90% wished to continue home monitoring after the pilot: “It helps me feel more in control”, “I like to monitor my own disease and be monitored” and “I hope this program can replace outpatient clinic visits in the future”. Daily home monitoring did not lead to higher anxiety levels (HADS anxiety score at baseline 4.5, score after 4 weeks 4.3, *p* = 0.57), and quality of life remained stable (K-BILD total score at baseline 59.2, score after 4 weeks 60.3, *p* = 0.65). Table 1 provides a comprehensive overview of potential barriers, experiences and solutions for use of the home monitoring system.

**Table 1:** A comprehensive overview of the identified potential barriers for use of the home monitoring system (wireless and real-time), experiences from the pilot study, and possible solutions as suggested by patients and staff

Potential barriers for the use of real-time home spirometry	Findings in our pilot experiment	Possible solutions
No internet access	Patient who never used internet before had no problems using the tablet and perform spirometry because of the simple design.	- Provide patients with a smartphone or tablet with 4G SIM card during study to guarantee internet access - Use a simple application without too much information
Quality of measurements is difficult to control	All patients performed mostly good quality flow volume loops, which could be checked real-time.	- New wireless spirometers have automated quality control and provide advice to patients - Use a device that shows a flow volume loop accessible to patient and researchers to review quality
A handheld spirometer may be difficult to use	A few patients had to get used to handheld spirometry the first days. Only one patient had variable results, due to technical difficulties with the standard mouthpiece. After providing an additional mouthpiece the readings were comparable to hospital readings.	- Provide a clear instruction manual and good training at start of the study. Patients should be able to perform 3 good quality measurements with ≤150 ml difference in the 2 highest FVC’s. - Assess individual patients’ needs - Consider using an extra/other mouthpiece - Use a video consultation or clinic visit for refreshment training
Motivation	A 6 s countdown and FVC target value is always shown during a forced expiration. This motivated patients to blow as good and long as possible.	- Do not use an FVC of 100% predicted as target value as this might demotivate patients - Provide an individual target value for each patient and adjust target value during study if necessary
Home spirometry might induce coughing	Some patients mentioned more urge to cough compared to hospital spirometry, but one measurement a day was not a problem at all.	- Advise patients to perform spirometry after a period of rest - Advise patients to try again later that day when a measurement failed because of coughing
Patients might get worried seeing their own results	Anxiety and depression scores were not higher after this short pilot. Almost all patients considered it pleasant to see their daily results.	- Incorporate automated email alerts to the researchers and explain to patients that they will be contacted if FVC declines significantly - Provide an extra option that blinds patients from their results
Daily home spirometry can be bothersome to patients	None of the patients in the pilot considered once daily spirometry bothersome, because it was not time consuming and became part of their routine.	- Advise patients to perform spirometry at almost the same time every day to create a routine - Explain that the whole process takes less than two minutes
Compliance	Patients got motivated by keeping track of their own results and almost all patients continued home spirometry after the pilot.	- Send patients email reminders when they do not perform spirometry or report their symptoms

## Discussion

This pilot study shows that a home monitoring program integrated with real-time wireless home spirometry is feasible in patients with IPF. In line with other studies, home-based measurements were slightly lower than hospital-based FVC, which may partly be equipment-related, but also effort-related [2, 4]. We tried to minimize the risk for ‘underperforming’ at home by motivating patients through graphically displaying their personal target value and prior results, a six seconds countdown and advices to technically improve the measurements. However, home and hospital readings are highly correlated and the relative variability of home-based FVC is low, indicating that home spirometry is a reliable tool to monitor patients at a distance. In a patient population with progressive breathlessness and decreasing mobility this enables close monitoring, while lowering the burden of hospital visits, especially in countries with long distances to the hospital. Moreover, real-time uploading of results and automated email alerts not only allow quality review of measurements, it also enables real-time detection of FVC decline. For example, we already observed a decrease in FVC two days before a patient reported symptoms of a respiratory tract infection. Early detection may potentially improve efficiency and quality of care for patients. Besides spirometry, patients also recorded symptoms and validated questionnaires online, which could be important additional features for future studies.

All patients in our study supported the usefulness of home monitoring, and appreciated being actively involved in monitoring their disease. One patient experienced technical problems with spirometry, highlighting the importance of good instruction. No effects on anxiety or quality of life were observed, however, we believe that the duration of the study is too short to draw definite conclusions on this. We found no major barriers regarding use of real-time wireless home spirometry; relatively easy solutions were suggested by patients and investigators for potential issues.

A limitation of this study is that it is a single center study, with 10 out of 12 consecutive patients willing to participate. In the Netherlands, use of internet amongst elderly people is rather high, however, also in other countries internet use among people over the age of 65 is steadily growing [9]. With worldwide increasing internet use and technological advances, we envision that relatively simple and low-cost systems like this, will facilitate access to care and research for a wider group of patients, also in remote areas and lower socio-economic settings. Further limitations of this pilot are the small sample size and short duration. Although this was sufficient to evaluate reliability and potential barriers of a home monitoring program with real-time wireless home spirometry, larger studies are required to assess whether it improves care, allows for earlier detection of exacerbations, and enhances data collection in clinical trials.

## Conclusion

A home monitoring program including wireless home spirometry, is highly feasible and appreciated by patients with IPF, and enables real-time detection of change in FVC and PROs facilitating personalized care.

## Abbreviations

FVC: Forced Vital Capacity
IPF: Idiopathic Pulmonary Fibrosis
PROs: Patient Reported Outcomes
